# Seroepidemiological study of Q fever in domestic ruminants in semi-extensive grazing systems

**DOI:** 10.1186/1746-6148-6-3

**Published:** 2010-01-20

**Authors:** Francisco Ruiz-Fons, Ianire Astobiza, Jesús F Barandika, Ana Hurtado, Raquel Atxaerandio, Ramón A Juste, Ana L García-Pérez

**Affiliations:** 1 NEIKER- Instituto Vasco de Investigación y Desarrollo Agrario, Department of Animal Health, Berreaga 1, 48160 Derio, Bizkaia, Spain

## Abstract

**Background:**

Q fever, a worldwide zoonotic disease caused by *Coxiella burnetii*, is endemic in northern Spain where it has been reported as responsible for large series of human pneumonia cases and domestic ruminants' reproductive disorders. To investigate pathogen exposure among domestic ruminants in semi-extensive grazing systems in northern Spain, a serosurvey was carried out in 1,379 sheep (42 flocks), 626 beef cattle (46 herds) and 115 goats (11 herds). Serum antibodies were analysed by ELISA and positive samples were retested by Complement Fixation test (CFT) to detect recent infections.

**Results:**

ELISA anti-*C. burnetii *antibody prevalence was slightly higher in sheep (11.8 ± 2.0%) than in goats (8.7 ± 5.9%) and beef cattle (6.7 ± 2.0%). Herd prevalence was 74% for ovine, 45% for goat and 43% for bovine. Twenty-one percent of sheep flocks, 27% of goat and 14% of cattle herds had a *C. burnetii *seroprevalence ≥ 20%. Only 15 out of 214 ELISA-positive animals reacted positive by CFT. Age-associated seroprevalence differed between ruminant species with a general increasing pattern with age. No evidence of correlation between abortion history and seroprevalence rates was observed despite the known abortifacient nature of *C. burnetii *in domestic ruminants.

**Conclusions:**

Results reported herein showed that sheep had the highest contact rate with *C. burnetii *in the region but also that cattle and goats should not be neglected as part of the domestic cycle of *C. burnetii*. This work reports basic epidemiologic patterns of *C. burnetii *in semi-extensive grazed domestic ruminants which, together with the relevant role of *C. burnetii *as a zoonotic and abortifacient agent, makes these results to concern both Public and Animal Health Authorities.

## Background

Q fever is a worldwide distributed zoonosis caused by *Coxiella burnetii*, a ubiquitous Gram-negative bacterium that is able to infect humans and a wide range of animals, both aquatic and terrestrial [1-3]. Q fever is a polymorphic disease in humans with subclinic, acute and chronic forms [1,3]. Several groups in Spain have studied the clinical aspects and the distribution of the disease in different regions [5-7]. The disease seems to be more prevalent in northern Spain than in the central and southern regions of the country, and it is especially high in the Basque Country (northern Spain) where large series of human pneumonia cases due to *C. burnetii *have been reported [1,8]. It is proposed that *C. burnetii *is maintained in nature following two different cycles, the wild cycle in which ticks and wild animals are involved, and the domestic cycle, where ruminant and other animal species such as dogs and cats are the main reservoirs [1,3]. Nonetheless, the link between both proposed cycles is currently weakly known, especially because the domestic cycle has been considered the main source for human infection [9] and has therefore been the focus of most studies.

Clinical outcome of *C. burnetii *infection in domestic ruminants consists of abortion and stillbirths in sheep and goats while it causes infertility and mastitis in cattle [10]. No clinical signs are evidenced in non-pregnant animals. When abortion occurs, or even when ewes lamb normally, animals shed a high number of *C. burnetii *through placenta, vaginal fluids, faeces and milk [1,11,12]. Infectious abortion in domestic ruminants is a multi-etiologic process that causes important losses in the livestock industry. Furthermore, the zoonotic nature of most of the abortifacient pathogens is a cause for concern from the perspective of Public Health. While attention has been paid to most of these pathogens, such as *Brucella*, *Chlamydophila *or *Toxoplasma *in sheep, others like *C. burnetii *have not been routinely investigated in cases of abortion. The recent application of molecular techniques to study fetal tissues and placenta indicates that *C. burnetii *could be more prevalent than previously thought [13,14].

Serological surveys have been carried out in many countries to evaluate the distribution of *C. burnetii *in domestic ruminants. In some areas bovine seems to be the main reservoir [15-17] whereas in others, goats [18,19] or sheep [20-22] are the main domestic reservoir. However in other studies, serological surveys indicate that the different domestic ruminant species can play together a relevant role in the domestic cycle of *C. burnetii *[3,23-25]. In addition, different management systems showed different degree of exposure to infection [26]. Regarding Spain, few serological evidences have been reported both in domestic and wild ruminants [5,27].

Under the current panorama of Q fever re-emergence and the lack of information on the current status of *C. burnetii *infection in domestic ruminants in one of the most traditional livestock producing regions in Spain, we aimed to determine the epidemiological situation of this pathogen in the framework of a larger project on coxiellosis in livestock and wildlife. This study updates the status of this zoonotic pathogen in the ruminants population of the Basque Country and highlights the main risks for human infection in a hyperendemic Q fever region. We also evaluated if the age of the animals might account for differences in exposure to *C. burnetii*. Moreover, relationships between *C. burnetii *contact rate and abortion history at the herd level were also investigated.

## Methods

### Study area

**Figure 1:**
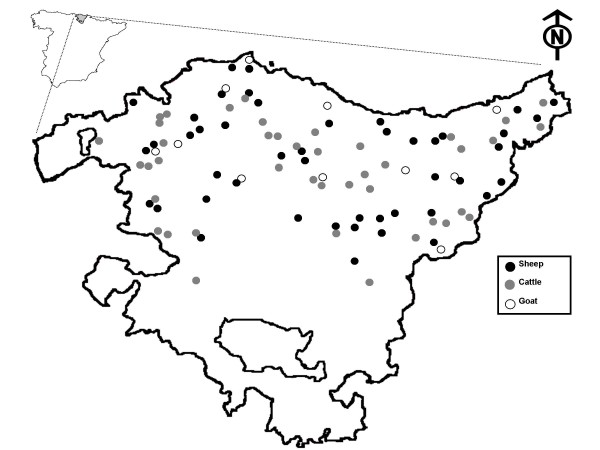
**Geographic distribution of the sampled herds in the study area**.

The Basque Country (7,234 km^2^) is a region located in northern Spain (Figure 1). Livestock production is one of the pillars of the rural economy of the region, with a total amount of 344,288 sheep, 160,000 cattle (dairy and beef) and 28,500 goats [28]. Livestock production is widely scattered throughout the region and the number of herds is high, with approximately 5,300 sheep, 6,915 cattle (dairy and beef) and 2,000 goat herds. Semi-extensive production is predominant for sheep, beef cattle and goats, and it is characterized by housing in winter months until early spring, when parturition occurs, and extensive grazing the rest of the year.

### Sampling approach

The survey was carried out from October 2007 to April 2008. Overall regional census was compiled for each ruminant species (data from the Basque Statistics Institute) and sample size was proportionally calculated according to the number of herds per county with a maximum number of 2,000 animals from 100 herds. Individuals (females) older than one year were randomly selected within herds to a maximum of 30 sheep, 15 beef cattle and 10 goats per herd. Finally, 1,379 sheep, 626 beef cattle and 115 goats from 46, 42 and 11 herds, respectively, were surveyed (Figure 1). Whole blood extractions were carried out by the veterinary practitioner groups in charge of the Official Sanitary Campaigns in the Basque Country, directed and supervised by the local Animal Health and Welfare Authorities (Diputaciones Forales).

After individual collection of blood into tubes without anticoagulant, sera were obtained by centrifugation and stored at -20°C until serological analyses were performed. History of recent abortion (*i.e*., occurrence of abortions during the two previous breeding seasons) was obtained from a number of the surveyed herds by interviewing the farmers during sample collection.

### Serological analyses

Sera were tested for the presence of anti-*C. burnetii *antibodies by means of an ELISA test (ELISA Cox kit, LSI-Laboratoire Service International, Lyon, France) according to manufacturer's instructions. Sensitivity of this ELISA test reaches 100% whereas specificity was determined to be of 95% (manufacturer's data). ELISA positive sera were retested for anti-*C. burnetii *antibodies by means of the Complement Fixation test (CFT) following standard procedures [29]. *C. burnetii *antigen was provided by Dade Behring (Marburg, Germany) using Nine Mile and Henzerling strains produced on embryonated eggs. According to the standards of the OIE, samples with CFT titres ≤ 1/5 were considered negative. Titres between 1/10 and 1/40 were representative for latent infection while titres ≥ 1/80 revealed an evolutive phase of the infection [29].

### Statistical analyses

For statistical analysis purposes, two age categories were established for each animal species (Table 1) in relation to the age at first parturition. Sheep and goats were classified in two groups, yearlings (1-2 years old) and adults (>2 years old), while beef cattle was classified as heifer (1-3 years old) or adult (>3 years old). Several animals younger than 1 year-old (73 sheep, 8 cattle and 6 goats) were sampled but were not considered for age-associated seroprevalence pattern analyses because of the smaller sample size.

**Table 1:** Mean anti-*C. burnetii *antibody prevalence (Serop.) and its associated standard error (SE) throughout ruminant species-by-age class.

	Age class	N	Pos	Serop. (%)	SE
Sheep	1-2 yr.	225	21	9.3	0.02
	Adult	1073	139	13.0	0.01

*Subtotal sheep*		*1298*	*160*	*12.3*	*0.01*

Beef cattle	Heifer	97	6	6.2	0.02
	Adult	521	35	6.7	0.01

*Subtotal cattle*		*618*	*41*	*6.6*	*0.01*

Goats	1-2 yr.	40	1	2.5	0.03
	Adult	69	8	11.6	0.04

*Subtotal goats*		*109*	*9*	*8.3*	*0.03*

N: Number of animals analyzed; Pos: Number of seropositive animals.

ELISA anti-*C. burnetii *antibody prevalence was calculated at individual and at herd level for each ruminant species. Rogan-Gladen correction (RGC) according to the sensitivity and specificity of the ELISA test was used for population seroprevalence calculation [30]. A herd was considered positive when at least one animal showed antibodies by ELISA. Thus, differences on antibody prevalence (continuous) between ruminant species, age classes (categorical) and abortion history (categorical, reported/non-reported) were statistically assessed by means of Chi-square tests. Statistical uncertainty was assessed by calculating the 95% confidence interval for each of the proportions according to the expression S.E._95%C.I. _= 1.96 [p(1 - p)/n]^1/2 ^[31].

## Results

ELISA test showed an average *C. burnetii *seroprevalence of 11.8 ± 2.0% (RGC: 7.1 ± 1.4%) in sheep, 8.7 ± 5.9% (RGC: 3.9 ± 3.5%) in goats and 6.7 ± 2.0% (RGC: 1.8 ± 1.0%) in beef cattle. Statistically significant differences were found between cattle and sheep seroprevalences (χ^2 ^= 11.7, *p *< 0.001). The percentage of seropositive herds was 74% (34/46) for sheep, 43% (18/42) for cattle and 45% (5/11) for goats. Within-herd seroprevalence values ranged 0-80% for sheep, 0-53% for cattle and 0-30% for goats, with 21%, 14% and 27% of the herds, respectively, showing a *C. burnetii *seroprevalence ≥ 20%.

Eight out of 162 (4.9 ± 3.3%), 6 out of 42 (14.3 ± 10.6%) and 1 out of 10 (10.0 ± 18.6%) ELISA-positive sheep, cattle and goats, respectively, also reacted positive in the CFT giving titres ≥ 1/10. CFT positive ewes belonged to 5 herds (5/46, 8.7%) and antibody titres did not exceed 1/20. In cattle, CFT positive animals belonged to 3 herds (3/42, 7.1%) and antibody titres did not exceed 1/40. Finally, only one goat reacting positive in ELISA also reacted in the CFT showing a titre of 1/10.

ELISA seroprevalence-age associated patterns differed between ruminant species (Table 1). While *C. burnetii *seroprevalence tended to increase from yearlings to adults in sheep and goats, seroprevalence values remained similar for heifer and adult cattle. Nonetheless, no statistically significant differences between age class seroprevalence values were found. In addition, anti-*C. burnetii *antibody prevalence did not differ statistically in relation to herd abortion history, neither in sheep (9.1 ± 3.9% for flocks reporting abortion and 9.0 ± 2.0% for non-reporting flocks) nor in cattle (10.7 ± 7.8% and 9.4 ± 3.9%, respectively). Seven of the 23 interviewed sheep flock owners reported recent problems of abortion. Five of these flocks were positive for ELISA anti-*C. burnetii *antibodies, with seroprevalences ranging between 3.3% and 36.7%. Interestingly, CFT titres ≥ 1/10 were detected in only one of those flocks. Meanwhile, 5 of 18 cattle herds reported abortion problems and showed seroprevalences between 0% and 20%, but none of the ELISA positive animals from those herds reporting abortion had significant CFT titres. Conversely, 6 of 13 cattle herds reporting no reproductive problems showed seroprevalences between 6.6% and 53.3% by ELISA, and in 2 of these herds several animals showed titres ≥ 1/10 against *Coxiella *by CFT. Recent abortion history could only be obtained from a small number of goat herds (n = 4) and consequently we did not considered them in the analysis.

## Discussion

Q fever is considered a strongly endemic disease in humans in the Basque Country [32,33]. Contact with domestic ruminants was considered one of the most relevant risk factors for *C. burnetii *infection. Moreover, *C. burnetti *was found to be widespread in sheep flocks [34] and is known to be a relevant agent for ovine abortion [13] that causes important economic losses to livestock producers. Nevertheless, very scarce information on the current status of this pathogen in other ruminant species, such as cattle or goats, was available for this region. As a first approach to improve knowledge on the current status of *C. burnetii *among livestock in the Basque Country, we studied the seroprevalence in beef cattle, sheep and goats, which are reared in semi-extensive conditions, while intensively managed species (dairy cattle) will be the subject of a future study.

Comparison of herein reported ELISA results with other epidemiological surveys has to be carefully considered due to the different serological techniques employed. While recent studies tend to use the indirect fluorescence assay (IFA) or ELISA, those carried out some decades ago used mainly CFT. Whereas IFA and ELISA tests on ruminant sera showed similar results, low agreement was observed between ELISA and CFT [35,36], which agrees with our observations in sheep (unpublished data). CFT has a good specificity but a low sensitivity [29]; in fact, only 15 out of 214 ELISA positive animals gave CFT titres ≥ 1/10. The highest sensitivity associated to the ELISA test together with the high duration of detectable levels of circulating anti-*C. burnetii *antibodies were the reasons for using this technique for epidemiological purposes in the current study. Although ELISA test can fail to detect *C. burnetii *contact at an individual level [36], it becomes very useful for studies at the population level like the one presented herein. On the other hand, molecular methods are highly sensitive tools for detecting *C. burnetii *infected animals. Nonetheless, the high variability of *C. burnetii *excretion by animals throughout the year limits the reliability of molecular methods for epidemiological purposes, especially if sampling takes place outside of the reproductive season.

The seroprevalence found in sheep was comparable to that reported in other Spanish regions where seroprevalence ranged between 1.7% and 18.8% [5] or in some Mediterranean regions, with values between 9% and 25% [19,22,24,37,38]. These data highlight the risk of *C. burnetii *zoonosis associated to sheep in all the Mediterranean countries. Nevertheless, seroprevalence of cattle and goats in some of these countries suggest that these species would also represent a potential risk. In fact, cattle seropositivity ranged between 5.8% and 25% [24,37], and in goats between 13% and 51% [19,24,38]. In Spain, the highest prevalences found in these species were 66.9% for cattle and 32.7% for goats in different production systems from central and southern Spain, respectively [5], and more recently, 35% of southern Spanish cattle in extensive production have been found to have contact with *C. burnetii *[27].

The lower seroprevalence observed in the present study in cattle and goats could be explained by the semi-extensive management conditions in which animals are moving during part of the year in large land surfaces thus reducing the contact between animals. Therefore, the different prevalences of *C. burnetii *in livestock suggest that local productive system and management factors might influence the life cycle of *C. burnetii*. Moreover, wildlife could play a relevant role as reservoir for livestock [27], especially where extensive productive systems are predominant, which increases the risk of wildlife/livestock contact. The significantly higher seroprevalence observed in sheep with regard to beef cattle could not be explained by differences in the sampling dates in relation to the lambing/calving season. Both beef cattle and sheep were surveyed coinciding with the end of the gestation period, when the excretion of *C. burnetii *by infected animals is higher because of parturition [10]. Interestingly, when CFT was analysed in ELISA positive animals, beef cattle had a higher percentage of reactors (14.3%) compared to sheep (4.9%) indicating a higher number of latent infections in cattle than in sheep. Nevertheless, the small size of the sample and the concentration of CFT-positive animals in a reduced number of herds make it difficult to reach a definitive explanation. Other management factors, like closer contact among ewes during milking, which implies overnight housing, could be the reason for a higher overall ELISA seroprevalence in sheep than in cattle.

Age-related *C. burnetii *serological patterns have been seldom reported in the scientific literature in regard to domestic ruminants [24,34]. Pathogen contact rate tends to increase with age simply as a consequence of a higher probability of contact with life span, a feature that herein was observed for sheep and goats.

Long-time contact with *C. burnetii *in the surveyed herds together with the random selection of sampled animals could explain the lack of association found between herd seroprevalence and abortion reports. Different results have been reported in this regard, with significant associations between seroprevalence and abortion reported for cattle and sheep in several studies [16,37], whereas in other reports infection with *C. burnetii *in cattle did not result in abortion, suggesting that infection sometimes can pass unnoticed [39]. In the current study, in 6 cattle herds without reproductive problems within-herd seroprevalence by ELISA ranged between 6.6 and 53% and in 2 of these herds several animals had CFT titers ≥ 1/10 indicating a recent contact with *C. burnetii*. Although several farmers did not report abortion or reproductive problems, high seroprevalences can be associated with the presence of *C. burnetii *in the herd. Similarly, Astobiza et al. [12] found a high within-flock seroprevalence (54%) and a high percentage of *C. burnetii *shedders at lambing (55-79%) in a sheep flock with a low rate of abortion (3.4%). These observations indicate that there are several epidemiological and clinical aspects of *C. burnetii *infection that need to be elucidated and require further investigation.

## Conclusions

The results reported herein showed that sheep had the highest contact rate with *C. burnetii *in the region, but also that cattle and goats should not be neglected as part of the domestic cycle of *C. burnetii*. Based on our observations, we can conclude that measures are to be implemented for the control of *C. burnetii *and Q fever in the study region along the line of those currently in operation in other European countries. Moreover, further epidemiological research on herd, local and regional factors influencing *C. burnetii *life cycle is needed in order to establish more efficient control measures that prevent spread of the infection and its associated effects on livestock and humans. At present, a control programme based on vaccination using a Phase I vaccine is in progress and its efficacy will be assessed in the near future.

## Authors' contributions

FR was responsible for data collection and statistical analysis, and wrote the manuscript; JFB, JA and RA helped in the samplings and performed the serological tests; AH assisted with the interpretation and discussion of results and correcting the manuscript; RAJ advised on statistical analysis and critically revised the manuscript. ALG is head of the project and had primary responsibility for the investigations reported here. All authors revised the manuscript and approved it in its final version.
